# Biomechanical Exposure to Upper Extremity Musculoskeletal Disorder Risk Factors in Hospital Laboratories

**DOI:** 10.3390/ijerph19010499

**Published:** 2022-01-03

**Authors:** Jung-Keun Park, Jon Boyer, Laura Punnett

**Affiliations:** 1 Department of Work Environment, University of Massachusetts Lowell, Lowell, MA 01854, USA; jboyer5@bwh.harvard.edu (J.B.); Laura_Punnett@uml.edu (L.P.); 2 Occupational Safety and Health Research Institute, Korea Occupational Safety and Health Agency, Ulsan 44429, Korea; 3 Department of Environmental Affairs, Brigham and Women’s Hospital, Boston, MA 02115, USA; 4 Department of Biomedical Engineering, University of Massachusetts Lowell, Lowell, MA 01854, USA

**Keywords:** ergonomics, exposure assessment, hospital laboratory, laboratory work, PATH method

## Abstract

Exposure to ergonomic risk factors has been reported for laboratory workers over decades. However, these exposures are not well characterized with respect to the type of laboratory or work organization. This study compared biomechanical exposure to upper extremity (UE) postures and hand activity levels (HALs) in general hospital laboratories by job, work, and laboratory type. The study used observational data gathered using a revised version of the Posture, Activity, Tools, and Handling (PATH) method, generating frequencies of categorized exposures. Eighteen workers were observed in 11 job titles (seven laboratories) in a single hospital by two investigators over a 7 month period. A taxonomy was constructed to categorize the extent to which the laboratory operations were automated. Overall, there were markedly high exposures to postural strain for the distal UE, especially wrist/forearm deviation (73% of observations), gross grasp (71%), and pinch grip (49%). For the HAL categories, 61% of the observations were in the moderate range (3.3–<6.7). Shoulders and elbows tended to remain in the neutral postural range. Posture frequencies were similar among the job categories studied and laboratory types. HAL was higher when the hand was in a pinch grip. Manual operations represented a higher proportion of work time than semi-automated or automated operations. Biomechanical exposure can be documented more extensively and diversely when using the revised PATH approach along with the taxonomy, with respect to exposure variables, such as the type of job, work, or organization in the industry including the hospital laboratories.

## 1. Introduction

Hospital laboratory work is primarily composed of the performance of various clinical tests on patient specimens (e.g., blood, urine, or tissue). As these tests often involve ergonomic risk factors, such as repetitive or prolonged hand activities, non-neutral upper extremity postures, and forceful exertions, laboratory workers are exposed to the risk factors for upper extremity musculoskeletal disorders (UEMSDs) [1,2,3,4].

With respect to MSDs in laboratory work, an incidence rate of 42.0 per 10,000 full-time workers for repetitive trauma disorders (RTDs) in medical and dental laboratories was reported to be about twice higher than the average rate (i.e., 23.8) in the US private industry in 2001 [5]. The prevalence of UEMSDs was documented for laboratory workers by numerous studies: the musculoskeletal discomfort prevalence was generally highest for the shoulder than for the wrist and the lowest for the elbow [2,3,6,7]. Pathologists [8] were shown to have more shoulder problems than pipette users [3]. Overall, the prevalence rates of UEMSDs were reported to be up to 60.2% [8], which was also supported by a review study, where the overall prevalence rates ranged from 40% to 60% [1].

Researchers have assessed the association between UE musculoskeletal health and ergonomic exposures in laboratory technicians [3,4,8]. One important limitation of such epidemiologic studies was a potential bias in self-reports of exposure to ergonomic risk factors. This limitation in exposure quantification is mitigated with direct observational techniques.

PATH (Posture, Activity, Tools and Handling), a direct observation technique based on work sampling, has been used for semi-objective characterization of ergonomic exposure in different industries such as construction [9,10,11,12,13], dairy farms [14], retail stores [15], fruit harvesting [16], healthcare [17,18,19,20], hotels [21], and fishery [22]. The PATH method can be used to characterize ergonomic exposures in terms of job task, time (e.g., day-to-day variability), and individual worker [9,10,12,13,19,23]. A revised PATH method was developed for healthcare workers to facilitate the quantification of specific risk factors, including UE postures and repetitive hand activity [17], and the work sampling method was shown to be reliably used in the assessment of exposures to a set of physical risk factors associated with MSDs among workers in hospital work [17] as well as construction [10].

A taxonomy, a hierarchical classification system, has been used for ergonomic exposure assessment by researchers. It was used to characterize the work hierarchically in exposure assessment and intervention studies in construction [24] and to systematically categorize nurse work into small work elements (e.g., tasks or activities) in general hospitals [25]. Moreover, the taxonomy was employed as a framework for the PATH methodology to describe the process of work in construction [10]. It is likely that the taxonomic approach, along with the revised PATH method, can classify laboratory work systematically in hospital laboratories.

Laboratory workers’ ergonomic exposures have been documented by a variety of factors, as aforementioned, including biomechanical factors for many years. However, such exposures are not characterized with respect to exposure variables such as the type of job, work, or organization. Given that job task analysis is generally undertaken prior to exposure assessment, it is quite useful to conduct ergonomic job task analyses using a taxonomy, which can provide information on work and organization [10,24,25]. Since modern trends in hospital laboratory work are towards multi-skilling, ergonomic exposures need to be explored more systematically or extensively in relation to a job’s, work’s, or an organization’s features in order to compute exposure profiles and identify opportunities for job/work redesign. Although those exposure variables are often used to organize information on ergonomic exposures in epidemiologic studies [3,9,26], based on the literature sought in this study, currently, there is still a lack of information on how such exposure variables are associated with ergonomic exposures.

In this respect, the objectives of this study were to assess biomechanical exposures to UEMSD risk factors using the revised PATH method in hospital laboratories and to examine those exposures more extensively and diversely using that method approach along with a taxonomy, with respect to job category, hand activity work type, and laboratory type in hospital laboratories.

## 2. Methods

### 2.1. Study Site and Subjects

This study was part of the epidemiologic study conducted by the Promoting Healthy and Safe Employment (PHASE) in Healthcare project team at the University of Massachusetts Lowell (UML) [17,27,28,29,30]. It was carried out at the laboratory department of a hospital in northeastern Massachusetts, USA. The department consisted of seven laboratories: Specimen processing (Lab I); Laboratory chemistry (Lab II); Hematology (Lab III); Blood bank (Lab IV); Microbiology (Lab V); Pathology (Lab VI); Administration and laboratory support services (Lab VII).

The laboratory department had, on average, 70 workers who were 50 (38 for 1st shift, 9 for 2nd, and 3 for 3rd) during weekdays and 20 (11 for 1st, 6 for 2nd, and 3 for 3rd) on weekends. Among them, 18 full-time laboratory workers with 11 job titles were selected from all shifts: The 18 (15 female and 3 male) subjects’ ages ranged from 22 to 62, and all but two of the workers, one each from the 2nd and 3rd shifts, worked on the 1st shift. The subjects were requested to permit investigator observation while they performed their usual jobs. Participation was completely voluntary and restricted to those with no musculoskeletal disorders or injuries during the past 12 months prior to the observation date. Each subject signed an informed consent form, and the entire study was approved by the UML Institutional Review Board.

### 2.2. Job Documentation

Written job descriptions and an organizational chart from the hospital provided information on workers’ qualifications, physical demands, work environment, organizational structure, and operational work processes in general for all seven laboratories.

The laboratory department had a total of 20 job titles which were associated with the 70 laboratory workers. Of the 70 laboratory staff, Lab Assistant was the largest group (27%), followed by Clinical Scientist II (23%), Clinical Scientist I (17%), Data Processing Clerk (8%), and others (25%).

Laboratory Supervisors provided technical and administrative supervision over some laboratory sections or shifts. The supervisors provided performance testing in compliance with standards. They developed and upheld departmental policies and also implemented changes to promote patient care.

Professional scientists largely consisted of Lead Clinical Scientists and Clinical Scientist II. Lead Clinical Scientists assisted with the technical functions of a laboratory section. They conducted clinical testing and related technical operations. Clinical Scientist II collected specimens from patients and performed a wide variety of test procedures.

Technical scientists mainly consisted of Clinical Scientist I who acted with the supervision of the Laboratory Supervisors or professional scientists to perform a variety of test procedures. On a daily basis, they conducted instrument maintenance and quality control testing according to established procedures. They collected blood and other patient samples for laboratory testing as well.

The support personnel consisted of support services and administration staff, including Lab Assistant, Support Services Leader, Lab Stock Technician, Data Processing Clerk, and Coordinator. Lab Assistants, for example, performed blood collection and specimen delivery to the laboratories.

The hospital laboratory jobs were classified into three job categories based on the ’“2018 Standard Occupational Classification (SOC) System [31]” and the “EEO 2014–2018 Occupation Crosswalk to other Occupations Groups [32]”: Professional, technician, and support worker. Professional and technician are defined as those who primarily conduct clinical tests or examinations for patient specimens: a professional is one who has at least a BA (Bachelor of Arts) or BS (Bachelor of Science) degree, while a technician is one who has two years of college courses or an equivalent education. A support worker is defined as one who conducts administrative or supportive work in the hospital laboratory department. In this study, supervisors and professional scientists were classified as professional, and a technical scientist was classified as a technician. Support staff members were classified as support worker.

### 2.3. PATH Data Collection

Electronic versions of three PATH templates were created using the InspectWrite™ software (Penfact Inc., Boston, MA, USA) and loaded onto a personal digital assistant (PDA) (Toshiba, Pocket PC e310 or e410, Tokyo, Japan) to record observations onsite. A stopwatch or digital watch was used to ensure fixed-interval sampling at standardized time intervals. Prior to every observation period, the PDA was fully recharged, and the watch was checked.

Two observers who completed a 30 h PATH training program individually collected PATH data in the laboratory department. On each observation day, a walkthrough and interview were first performed in the work area where each subject was to be observed for the day. A laboratory work checklist, which was developed for this study, was used to collect further information such as the characteristics of job title, laboratory type, amount of time spent on work operations, hand activities in conjunction with observed tasks (e.g., pipetting or sample preparation), and equipment or instruments in use. A brief discussion was held with the worker during downtime or breaks. The checklist was primarily filled out during the walkthrough and interview.

Observations were made over a 7 month period. An observation was defined as one complete set of all items on the three PATH templates. The observations were made at intervals of 90 s. An observation period was defined as a work shift during which a set of PATH observations was collected from one subject for up to several hours, depending on subjects’ availability. During PATH observation, each item was assumed to be independent of other items. For each item of the whole body template (e.g., shoulder/arm or elbow), the highest (most severe) exposure observed during the first 45 s of the observation was recorded. The next 30 s of observation were dedicated to the hand/forearm template, which was used to code exposures for the dominant hand. The last 15 s of observation were used for evaluating repetitiveness and the pace of hand activity and recording a hand activity level (HAL) value [33,34]. The HAL was scored as an integer from 0 to 10 and then input into the hand activity template. More information on the data collection procedures using this version of the PATH method is described elsewhere [17].

### 2.4. Data Management

PATH data were transferred into the authoring workstation (i.e., personal computer) from the PDA. The data were visually reviewed for errors (e.g., typos), manually cleaned, and stored for future analyses right after observation. Of the 18 PATH items (ergonomic risk factors) collected for each observation, those used for this study were HAL and 5 non-neutral UE postures, i.e., shoulder/arm elevation, elbow posture, wrist/forearm deviation, neutral/gross grasp, and pinch grip.

### 2.5. Data Analysis

For tabulation analyses, the recorded HAL value for each observation was categorized as slow (0–<3.3), moderate (3.3–<6.7), or rapid (6.7–10). Using the median of the continuous HAL values for each observation period, the work was classified as “low hand activity work” if the median was 0–<5.0 and “high hand activity work” if 5.0–10. Six workers were observed twice in different conditions such as date, laboratory type, and/or observer. Because so few observation periods were repeated from the same workers, they were treated as independent.

With the data gathered using the laboratory work checklist, we constructed a taxonomy to document the features of laboratory work in the hospital. Each laboratory task was broken into a series of work operations and each operation was composed of numerous work elements. An “operation” was defined as a process involving a defined group of tasks and/or activities to achieve a specified goal, whether performed routinely or non-routinely [24]. Along with supplementary information obtained from the facility documents (e.g., job descriptions and organizational chart) and interviews with the subjects, the taxonomy was reiteratively revised through discussion with a supervisor in the facility, two clinical scientists at university hospitals as ad hoc reviewers, and UML researchers who had experience in clinical laboratory work. In the hospital studied, specimen processing was administratively a part of laboratory chemistry, but it was treated as a separate laboratory in this study, since many hospitals consider it as an independent section. Similarly, the “night laboratory” was not included in the taxonomy because this was an administrative designation for a few personnel who performed any operations in the whole laboratory department during the 2nd and 3rd shifts.

The data included in the taxonomy were used to quantify the proportion of observed work time spent on each operation (i.e., %time) for each laboratory. The quantified estimates were averaged for each operation, assuming that the observed days were representative samples and that the subjects usually worked for 8 h per day. The laboratory operations were classified qualitatively in terms of the extent to which the work was performed manually vs. with automated equipment. “Manual” operations were those primarily processing individual samples or small batches, tasks performed with hand-held equipment, and hand-written or keyed-in data entry. “Automated” operations involved using electronic systems that processed large numbers of samples at a time, and which the operator could leave unmonitored for periods of time while performing other tasks. “Semi-automated” operations involved a mix of automated and manual tasks.

In each observation period, the frequency of each of the 6 ergonomic risk factors was estimated as its percentage of observations. For all observation periods, exposure was quantified as the mean percentage of observations, along with standard deviation in each of the risk factor categories. The mean exposure frequency estimates were compared using analysis of variance (ANOVA) or *t*-test with respect to job category, low/high hand activity level, and laboratory type. In the assessment of such exposure frequencies, the observation period was regarded as the unit of analysis and the significance level was 0.05. Data were analyzed using SAS 9.4 (SAS Institute Inc., Cary, NC, USA).

## 3. Results

A total of 24 PATH observation periods were obtained for each of the UE postures and HAL categories (Appendix A Table A1). Twenty-one of the 24 observation periods covered the worker’s right hand in the hand/forearm template. The observation periods were composed of 2165 observations for shoulder and elbow postures, and of 2186 observations for hand/forearm postures as well as HALs. The observation durations ranged from 105 to 380 min for the 24 observation periods.

In the seven laboratories, each laboratory consisted of two–six operations (Table 1). The mean percent time spent on each operation varied across laboratories (Table 2). Among the mean percent time data, the highest mean percent time was manual sample preparation (95 ± 0% time) in the laboratory pathology. Compared with the mean percent times spent on automated work operations, those spent on manual work operations were commonly higher, except in laboratory chemistry.

### 3.1. Exposure to Biomechanical Risk Factors

**Figure 1:**
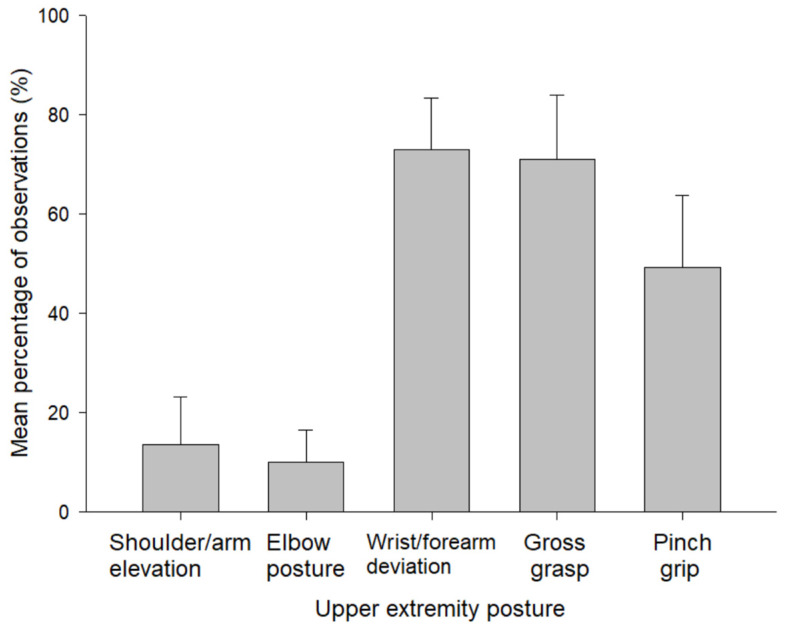
Mean percentage of observations, along with standard deviation (error bar), for each non-neutral upper extremity posture category: hospital laboratory employees (*n* = 24 observation periods).

**Figure 2:**
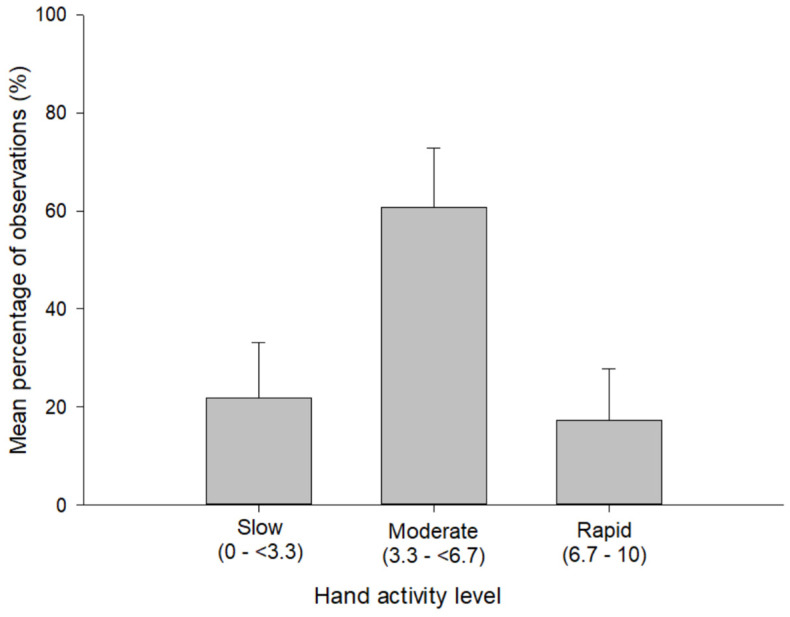
Mean percentage of observations, along with standard deviation (error bar) for each category of hand activity level (HAL) in the hospital laboratories (*n* = 24 observation periods).

The most frequent non-neutral posture was wrist/forearm deviation (73% of observations), followed by gross grasp (71%) and pinch grip (49%) (Figure 1). The exposure frequencies for non-neutral shoulder/arm and elbow postures were much lower: 13% and 10%, respectively. The overall median HAL value was 5.0 for the 2186 HAL scores, which were not normally distributed. The moderate range (3.3–<6.7) represented 61% of all observations, followed by 22% for slow (0–<3.3) and 17% for rapid (6.7–10) (Figure 2).

### 3.2. Comparison of Measures by Exposure Variable

**Figure 3:**
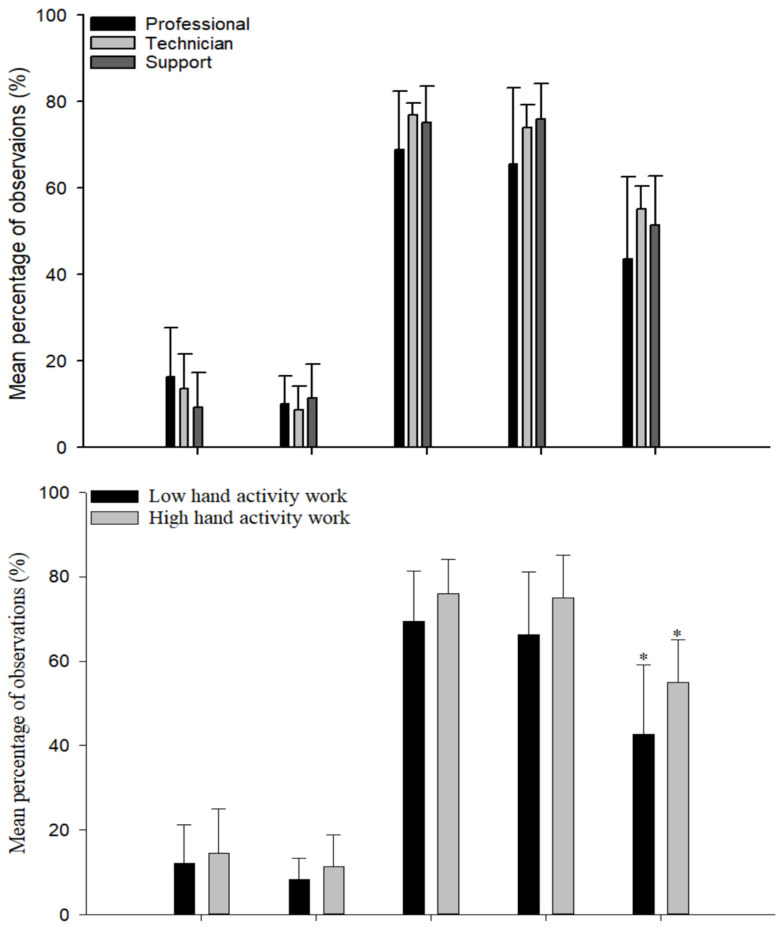

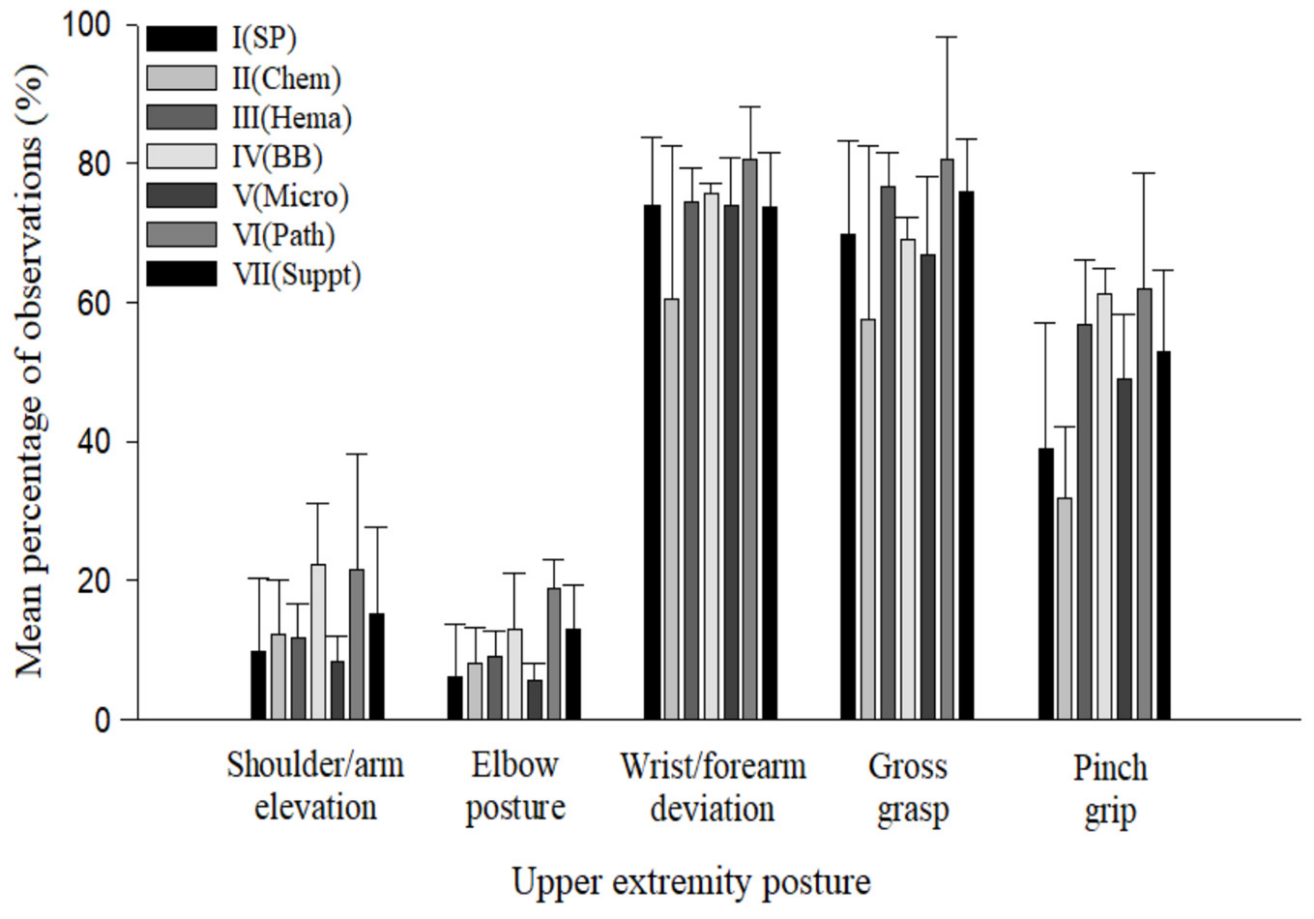
Mean percentage of observations, along with standard deviation (error bar), for each non-neutral upper extremity posture by job category (**top**), hand activity work type (**middle**), and laboratory type (**bottom**) (*n* = 24 observation periods; * *p* < 0.05).

**Figure 4:**
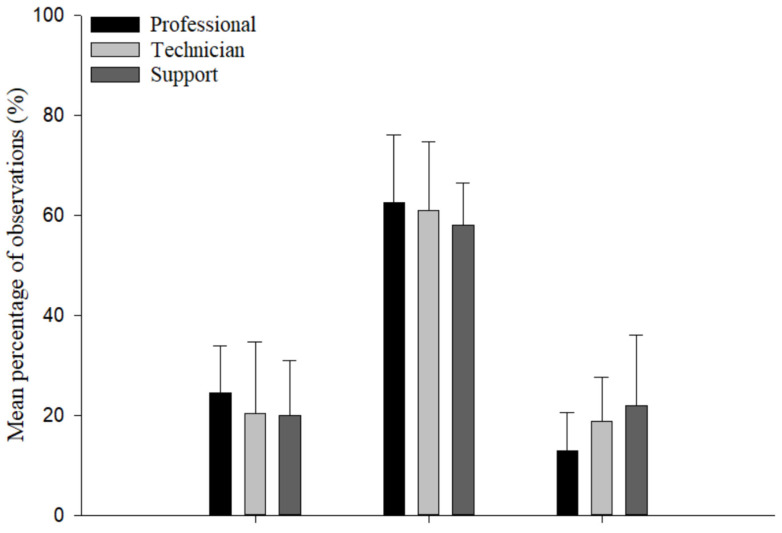

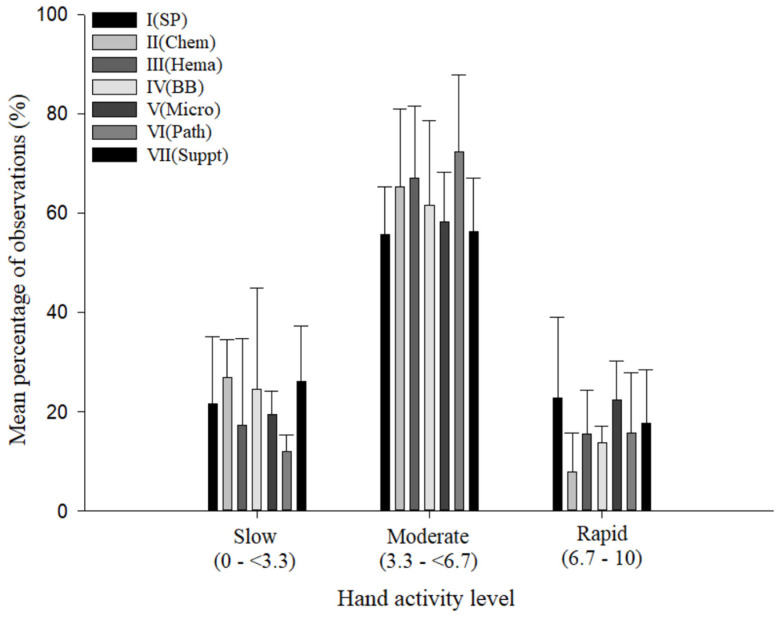
Mean percentage of observations, along with standard deviation (error bar), for each category of hand activity level by job category (**top**) and laboratory type (**bottom**) (*n* = 24 observation periods).

The mean percentage of observations in each of the UE postures was compared by job category, low/high hand activity, and laboratory type (Figure 3). Fourteen out of 15 sets of UE posture frequencies did not differ significantly (*p* > 0.05). However, exposure frequencies for pinch grip were significantly different between high and low hand activity work type (*p* < 0.05). Frequencies of the three HAL categories were not significantly different (*p* > 0.05) among job categories and laboratory types (Figure 4).

## 4. Discussion

### 4.1. Findings and Implications

For this study, 24 work shifts of 18 hospital laboratory workers were observed. Over 2165 90-s observations were completed, and the frequencies of six biomechanical risk factors were estimated. The laboratory workers performed hand activities or manipulative tasks with somewhat highly repetitive motions and awkward posture of the hands and fingers, while their shoulders and elbows remained in the neutral postural range.

Among the UE postures, the highest exposure frequency was 73% for non-neutral wrist/forearm posture. Pinch grip frequencies were significantly higher in hand activity work with higher HAL scores. Exposure frequencies were markedly high for distal UE postures, whereas shoulder/arm and elbow postures were more often close to anatomically neutral. More than 60% of the work shifts had a median HAL value in the moderate range (3.3–<6.7). Among the work operations performed in the hospital laboratories, the most frequent one was manual sample preparation (95 %time) in the laboratory pathology. Overall, more time was observed in manual rather than automated operations.

The discomfort prevalence of UEMSDs was generally highest for the shoulder (e.g., 60.2%) [8], followed by the wrist and then the elbow in different studies [2,3,6,7]. In this study, however, frequencies of exposure to non-neutral postures were highest for the distal UE (e.g., 73% for the wrist/forearm), followed by the shoulder/arm and the elbow. It was noticeable that the highest discomfort prevalence was in the proximal body part in the studies, whereas the highest frequency of biomechanical exposures was in the distal one in this study. This implies that exposure outputs in this study may not support those study results associated with health effects attributed to ergonomic factors in laboratory workers.

The laboratory jobs studied here presented generally similar biomechanical exposure profiles, at the level of detail obtained. Exposure frequencies significantly differed only for pinch grip between high and low levels of hand activity work. Among laboratory types, the exposure frequencies of distal UE postures and rapid HAL work were noticeably lower for laboratory chemistry (Figure 3 and Figure 4 (bottom)). These low-exposure frequencies were likely attributable to automated chemistry operations such as auto-pipetting or auto-injecting systems. This indicates that automation of manual work operations is one possible work redesign approach to reducing biomechanical exposure to UEMSD risk factors in the laboratory sector.

### 4.2. Exposure Assessment and Methodological Issues

In workplace surveys, one could perform two types of exposure assessments: (1) entire job analysis identifies jobs within a facility that may present risk factors; (2) in-depth analysis (e.g., task-by-task level) examines individual jobs or tasks to assess exposure to risk factors in much higher detail [35]. The entire job analysis approach can provide general features (global pictures) of ergonomic exposure for the jobs or occupations in the study population. Compared to the entire job analysis, the task-by-task analysis has the strength to characterize the exposures in much more specific levels of work elements such as tasks or activities. In other words, lower levels of work elements can be more favorably captured by the task-by-task rather than the entire job analysis approach.

The PATH instrument used in this study was designed to assess ergonomic exposure comprehensively for groups of jobs across multiple departments and healthcare settings within the context of a social epidemiologic investigation [27,28,29,30]. The PATH data are thus very detailed in some respects but limited in other ways. The study results facilitated comparison among a wide range of hospital laboratory jobs of exposure to biomechanical risk factors for MSD or injuries, methods such as heavy lifting, and other whole-body exertions that are rarely seen in laboratory departments. The trade-off was a lack of detail about the specific fine motor activities typically found in laboratory work, which might have pointed to whether individual tasks required redesign. However, this method probably approached the limit of what can be discerned by visual observation and manual recording, as the hand is capable of very quick motions that would be difficult for the human observer to count or characterize in real time.

The study features a large number of observations of hospital laboratory work over a 7 month time period. Task information was obtained from the observations as well as the taxonomy using the laboratory work checklist rather than a task-based sampling protocol. Further, the observations were made according to a sampling protocol that avoided synchronization with any work cycles. These features combined to provide an unbiased sample with a structure that is ideal for examining organizational sources of variability [36].

Video recording was not utilized for this study, because it requires at least double the assessment time and the budget constrained this effort. Among the resulting limitations, only the “worst case” shoulder/arm and elbow postures were recorded, meaning that less severe but still injurious exposures might have been overlooked. Forearm and hand exposure information was recorded only for the dominant hand. Continuous estimate of UE postures, such as wrist flexion/extension or radial/ulnar deviation, was not attempted; only dichotomized postures were recorded. Furthermore, information on exposure to psychosocial factors was not available in the subset data.

## 5. Conclusions

In the observed hospital laboratory jobs, the most frequent non-neutral UE posture was wrist/forearm deviation (73% of all observations). Hand activity level was most often “moderate”, involving steady motion and infrequent pauses. Exposure frequencies did not generally differ much by job category, hand activity work type, or laboratory type. However, pinch grip posture was significantly more common at moments when the hand activity was classified as high. The proportions of observed work time spent in repetitive work were higher in manual rather than semi-automated or automated operations. Overall, there were markedly high exposures to postural strain for the distal UE while moderately high exposures to hand activity in the laboratory work, indicating that the revised PATH method can be used for assessing biomechanical exposures and, in combination with a taxonomy, for characterizing those exposures more extensively and diversely with respect to exposure variables such as job category, hand activity work type, and laboratory type in the laboratory sector.

In order to effectively minimize musculoskeletal problems in laboratory work, equipment and process re-design should seek to reduce the specific exposures of frequent pinching, wrist bending, and repetitive motion. Although it might negatively affect employment opportunities, one way to reduce biomechanical exposures would be to change from manual operations to automated ones such as auto-pipetting systems. The revised PATH instrument is a feasible way to assess whether or not attempted control measures achieve reduction of biomechanical risk factors for UEMSD in general laboratory work.

## Figures and Tables

**Table 1:** Taxonomy of hospital laboratory work in terms of work operations in the hospital laboratories.

Laboratory Type	Operation	Description	Example Work Element
Specimen processing	Pre-sample processing	Specimens are received from different places including departments of a facility, local clinics (laboratories), and residents. Specimens are registered (each accession number is assigned).	Sample manual handling (reception; capping and decapping of specimen tubes); VDU (keyboarding for registration); opening specimen bullets (a delivery box); labeling/writing; phone.
	Sample processing	Samples are preliminarily prepared using instruments (e.g., centrifuge or stirrer) or tools (e.g., pipette) before delivery. Minor testing is performed.	Sample manual handling (capping and decapping of specimen tubes); pipetting; VDU (result recording; test report); instrument use or operation; labeling/writing; phone.
	Post-sample processing	Specimens are delivered to lab sections, using VDU to track specimens and make sure they have been properly handled.	VDU (tracking, result report); sample manual handling (delivery to lab sections); phone.
Laboratory chemistry	Automated chemistry	Placing specimens on the appropriate instrument according to the tests ordered.	Pipetting; instrument operating (sample analysis, result report); phone
	Manual chemistry	Like osmometry and acetone testing, specimens are tested manually. Blood and gas samples are analyzed.	Sample manual handling (sample preparation); pipetting; labeling/writing; phone.
Hematology	Automated hematology	Placing test tubes on the appropriate instrument for analysis.	Instrument operating (sample analysis, result report); sample manual handling (sample preparation); pipetting; phone.
	Manual hematology	CBC (complete blood count), differentials, and urine microscopy are performed. Dipstick urines are performed on a CLINITEK.	Sample manual handling (sample preparation); pipetting; microscopy (making test slides; microscope use); VDU (result report); Instrument operating (centrifuging; sample test), labeling/writing; phone.
Blood bank	Blood testing (type, cross match, HIV)	Blood type (ABO/RH) and cross-match are tested. HIV testing is conducted on semi-automated equipment.	Sample manual handling (sample preparation); pipetting; Instrument operating; VDU (record results, result report); labeling/writing; phone
	Blood bank database review	Record or review blood information in blood bank database (paperwork and statistics)	VDU (record or review database; check stock status).
Microbiology	Automated microbiology	Tests performed on instrumentation; some biochemical tests on bacteria.	Sample manual handling (sample preparation); instrument operating; pipetting; VDU (result review and report); phone.
	Manual microbiology	Agar plates are inoculated with various specimens and examined for the presence of bacteria; gram stains, ova, and parasite examinations are performed. Other work (immunology/serology) may exist.	Sample manual handling (inoculating culture plates; storage; opening and closing plates; reading plates); pipetting; microscopy (microscope use); labeling and writing; VDU (result review and report); phone.
Pathology (histology, cytology)	Automated sample preparation	Tissue samples are prepared as ordered for analysis by fixation or staining.	Sample manual handling (sample preparation; fixation; staining); instrument operating; VDU (result review and report); phone.
	Manual sample preparation	Sample preparation includes cutting and cover mounting. During preliminary examination, samples are handled on a microscope (gross examination may be conducted).	Sample manual handling (cutting tissues; cover mounting); microscopy (pre-examination); instrument operating; VDU (result review and report); labeling and writing; phone.
Administration and laboratory support services	Staffing	Workforce is managed for shifts and rotation work; supervision.	VDU (data review and report); phone.
	Planning and budgeting	Planning and budgeting for lab department; coordinating with other departments.	VDU (data review and report); phone.
	Phlebotomy	Blood is sampled from outpatients and inpatients by phlebotomists. Information of patients is recorded and reviewed in the databases.	Specimen sampling (e.g., drawing blood); sample handling (blood rocker); VDU (record or review patient information); phone.
	Specimen transport	Samples are delivered to the specimen processing section (manually or mechanically).	Carrying delivery box; lifting and lowering; preparing and loading specimens to the pneumatic transport system (“the bullet”).
	Material stock	Supply and storage of lab materials; inventory and ordering.	Push-pull carts; carrying lab materials; VDU (data review, material order, and report); phone.
	Data processing	Information on phlebotomy work or specimen transport will be entered at PCs.	Document and record review; VDU work; phone

**Table 2:** Mean percent time (% time) spent on each operation by laboratory type: seven laboratories in one US hospital.

Laboratory Type	Operation		%Time: Mean ± Standard Deviation **	Number of Observation Period (Work Shift)
	Name	Notation *		
Specimen processing	Pre-sample processing	M	47 ± 32	4
	Sample processing	SA	33 ± 31	
	Post-sample processing	M	10 ± 4	
	Others	M	10 ± 7	
Laboratory chemistry	Automated chemistry	A	85 ± 13	3
	Manual chemistry	M	-	
	Others	M	15 ± 13	
Hematology	Automated hematology	A	52 ± 48	3
	Manual hematology	M	40 ± 40	
	Others	M	8 ± 10	
Blood bank	Blood testing (type, cross match, or HIV)	SA	94 ± 6	2
	Blood bank database review	M	-	
	Others	M	6 ± 6	
Microbiology	Automated microbiology	A	-	4
	Manual microbiology	M	93 ± 12	
	Others	M	7 ± 2	
Pathology (histology or cytology)	Automated tissue preparation	A	-	2
	Manual sample preparation	M	95 ± 0	
	Others	M	5 ± 0	
Administration and laboratory support services	Staffing	M	5 ± 12	6
	Planning and budgeting	M	9 ± 22	
	Phlebotomy	M	25 ± 39	
	Specimen transport	SA	2 ± 4	
	Material management and stock	M	16 ± 41	
	Data processing	M	35 ± 47	
	Others	M	8 ± 8	
	Total			24

* M: manual; SA: semi-automated; A: automated. ** Mean percent time was estimated for an operation by averaging the proportion of observed work time represented by each operation in each laboratory.

## Data Availability

Not applicable.

## Appendix A

**Table A1:** PATH observations for non-neutral upper extremity posture and hand activity level categories by observation period in the hospital laboratories.

Observation Period (Work Shift)	Subject *	Whole Body Template			Hand/Forearm Template				Hand Activity Template				Obs. Duration, min
		Obs. No.	Shoulder/Arm Elevation, %	Elbow Posture, %	Obs. No.	Wrist/ Forearm Deviation, %	Gross Grasp, %	Pinch Grip, %	Obs. No.	Slow, %	Moderate, %	Rapid, %	
1	A	63	12	7	80	73	55	35	80	22	61	17	165
2	A	115	7	8	137	64	61	50	137	17	68	15	380
3	B	62	35	15	58	78	78	50	58	38	41	21	122
4	C	44	33	22	45	86	93	74	45	10	83	7	120
5	D	38	19	11	39	84	86	38	39	19	81	0	111
6	E	150	24	17	155	72	60	52	155	18	55	27	375
7	E	115	14	12	114	58	44	38	114	28	65	7	260
8	F	52	4	2	50	40	42	20	50	34	50	16	157
9	F	69	6	1	65	62	57	12	65	22	69	9	215
10	G	131	8	5	125	72	80	67	125	37	52	11	316
11	H	150	16	7	143	75	71	59	143	39	49	12	335
12	H	70	29	19	69	77	67	64	69	10	74	16	181
13	I	137	11	2	129	78	81	55	129	24	45	31	354
14	I	138	4	5	139	80	71	56	139	14	60	26	335
15	J	40	18	13	44	80	71	55	44	10	80	10	122
16	K	139	9	6	129	77	78	49	129	40	49	11	302
17	L	44	9	9	44	72	79	49	44	5	69	26	105
18	M	76	9	5	79	58	73	70	79	16	59	25	154
19	N	89	0	0	97	85	84	43	97	7	49	44	255
20	O	65	10	16	73	75	68	50	73	14	62	24	200
21	P	124	26	10	120	77	71	60	120	42	53	5	375
22	Q	113	9	17	111	76	66	35	111	23	58	19	259
23	R	63	3	8	65	74	87	48	65	21	74	5	153
24	R	78	8	23	76	80	81	55	76	16	52	32	190
Mean	-	-	13	10	-	73	71	49	-	22	61	17	-
Total	-	2165	-	-	2186	-	-	-	2186	-	-	-	-

* Six out of 18 subjects were observed twice (Job title of A: Supervisor Lab; B: Senior Lab Supervisor; C: Clinical Scientist Histology; D, E, F: Clinical Scientist II; G: Lead Clinical Scientist; H, I, J, K, L: Clinical Scientist I; M, N: Lab Assistant; O: Lab Pathology Assistant; P: Lab Stock Technician; Q: Support Service Leader; R: Lab Data Processing Clerk).
